# Performance of Regular and Modified Canola and Soybean Oils in Rotational Frying

**DOI:** 10.1007/s11746-013-2278-0

**Published:** 2013-06-01

**Authors:** Roman Przybylski, Eliza Gruczynska, Felix Aladedunye

**Affiliations:** Department of Chemistry and Biochemistry, University of Lethbridge, Lethbridge, AB T1K 3M4 Canada

**Keywords:** Frying stability, Canola oil, Soybean oil, Tocopherols degradation, Sensory assessment, Polar components, Thermo-oxidative degradation

## Abstract

Canola and soybean oils both regular and with modified fatty acid compositions by genetic modifications and hydrogenation were compared for frying performance. The frying was conducted at 185 ± 5 °C for up to 12 days where French fries, battered chicken and fish sticks were fried in succession. Modified canola oils, with reduced levels of linolenic acid, accumulated significantly lower amounts of polar components compared to the other tested oils. Canola oils generally displayed lower amounts of oligomers in their polar fraction. Higher rates of free fatty acids formation were observed for the hydrogenated oils compared to the other oils, with canola frying shortening showing the highest amount at the end of the frying period. The half-life of tocopherols for both regular and modified soybean oils was 1–2 days compared to 6 days observed for high-oleic low-linolenic canola oil. The highest anisidine values were observed for soybean oil with the maximum reached on the 10th day of frying. Canola and soybean frying shortenings exhibited a faster rate of color formation at any of the frying times. The high-oleic low-linolenic canola oil exhibited the greatest frying stability as assessed by polar components, oligomers and non-volatile carbonyl components formation. Moreover, food fried in the high-oleic low-linolenic canola oil obtained the best scores in the sensory acceptance assessment.

## Introduction

Frying is one of the most popular food preparation methods and consumption of fried foods has increased significantly in the last decades because they are fast to prepare, relatively cheap, with appealing typical flavor, golden brown color, and a crisp texture [1]. However, during frying, usually performed at elevated temperatures, the following chemical reactions, among others, occur: oxidation, hydrolysis, oligomerization, isomerization, and cyclization. As a result, changes in frying oil occur and a wide variety of degradation products are produced, including volatile components and non-volatile polar and oligomeric compounds [2]. The physical and chemical changes occurring during frying affect both the frying performance of the oil and the sensory characteristic of the fried food [2, 3].

The exposure of oils to oxygen during frying makes oxidation the most prevalent of all degradation processes [2]. Thus, the oxidative stability of oils and fats is one of the most important factors used in oil quality assessment. The susceptibility of frying oils to thermo-oxidative degradation have been related to the level of fatty acids unsaturation and several modifications to reduce the contributions of these acids, especially α-linolenic acid has been done. Partially hydrogenated fats offer better resistance to thermo-oxidative degradation, extending fry-life and until recently, were the main frying medium for industrial and institutional frying operations. However, in response to current *trans* fats labelling regulations, a new generation of frying oils with modified fatty acids and other constituents have been developed utilizing breeding, and interesterification, among others.

Several studies have examined the frying stability of modified oils [4–7]. Warner and Mounts [7] established that the level of linolenic acid can be a decisive factor describing frying performance of soybean and canola oils. However, these authors did not take into account potential effect of linoleic acids which may have similar destructive effect [8].

Przybylski and Zambiazi [9] using neural network system established that only 50 % of oil oxidative stability can be predicted using fatty acid composition as the defining factor. Other similar studies have also suggested that thermo-oxidative stability of oils and fats cannot be accurately predicted based solely on the fatty acid composition, other endogenous minor components play an important role in the oil degradation during frying [6, 10, 11]. However, many of these studies were conducted with French fries as the sole food product, or by heating oil in the absence of food. It has been observed that the types of chemical reactions taking place during frying are different from those happening during heating without food [2]. Whereas heating of oil without frying food is usually more destructive. Further, various components of the fried food are known to participate in the degradation reactions occurring during frying, affecting the overall performance of the oil [12].

The aim of the present research was to compare the frying performance of modified and regular canola and soybean oils during rotational frying of different food products, mimicking a typical institutional frying operation.

## Materials and Methods

### Oils and Foods

Commercially refined, bleached and deodorized, regular and modified canola and soybean oils were obtained from Richardson Oil Processing (Lethbridge, Canada). Five oils were used as follows: regular canola oil (CAN), high-oleic low-linolenic canola oil (HOLLCAN), canola hydrogenated frying shortening (HCAN), regular soybean oil (SOY), and hydrogenated soybean frying shortening (HSOY). The same batch of French fries par-fried in HOLLCAN; battered chicken and fish sticks par-fried in regular canola oil were used in the frying experiments.

### Frying Procedure and Sampling

The frying was performed at 185 ± 5 °C for 7 days using HCAN, HSOY and CAN oils and 11 days frying was applied for HOLLCAN and SOY oils. Frying was done in restaurant style stainless steel fryers (General Electric Company, New York, USA) using 3.5 L of oil which was replenished every second day with 500 mL of fresh oil. The oils examined were conditioned at 185 °C for 2 h prior frying. Par-fried French fries, battered chicken and fish sticks were fried in succession forming one rotational cycle and nine cycles were run daily in each oil. For the cycle, a batch of 400 g of each product was fried for the following times: French fries for 5 min, battered chicken for 7 min, and fish sticks for 7 min. Daily, 3.6 kg (8 lbs) of each product was fried in each oil giving a total of 10.8 kg (24 lbs) of food per frying day and a load of 3.1 kg (6.9 lbs) of food per 1 L of oil.

### Fatty Acid Composition

Fatty acids were methylated prior to analysis by gas chromatography (GC) based on the AOCS Official Method Ce 1–62 [13]. The resulting fatty acid methyl esters (FAME) were analyzed on a Trace GC Ultra gas chromatograph (Thermo Electron Corporation, Rodano, Italy) using a Trace TR-FAME capillary column (100 m × 0.25 mm × 0.25 μm; Thermo Scientific, Waltham, MA, USA). Hydrogen was used as carrier gas with flow rate of 1.5 mL/min. Column temperature was programmed from 70 to 160 °C at 25 °C/min and held for 30 min, then further programmed to 210 °C at 3 °C/min. Initial and final temperatures were held for 5 and 30 min, respectively. Splitless injection was used utilizing PTV injector. Detector temperature was set at 250 °C. FAME samples, 1 μL, were injected with AS 3000 autosampler (Thermo Electron Corporation, Rodano, Italy). Fatty acids were identified by comparison of retention data with authentic standards purchased from Nu-Chek-Prep (Elysian, MN). *Trans* isomers in the oils are quantified as a group and presented as the sum of all *trans* isomers of oleic, linoleic and linolenic acids.

### Polar Components (PC)

The total amount of PC was determined by the gravimetric method after column chromatography separation of non-polar fraction according to the AOAC Method 982.27 [14]. Two fractions were collected: (1) the non-polar one eluted with hexane:isopropyl ether (90:10 v/v); (2) the polar one removed with isopropyl ether : methanol (90:10, v/v). The last fraction was analyzed for composition.

### Polar Components Composition

The polar fractions obtained above were analyzed using high performance size exclusion chromatography (HPSEC) following the ISO Method 16931-2007 [15]. Separation was performed on a Finnigan Surveyor liquid chromatograph (Thermo Electron Corporation, Waltham, MA). Components were separated on three Phenogel columns connected in series (500 Å, 100 Å and 50 Å, 5 μm, 300 × 4.60 mm; Phenomenex, Torrance, CA), using tetrahydrofuran (THF) as the mobile phase at a flow rate of 0.3 mL/min, and columns held at 30 °C. A 10 μL sample was injected, and components were detected with a Sedex 75 evaporative light scattering detector (Sedere, Alfortville, France), operated at 35 °C with air pressure of 2.5 bar. Using this system the following compounds were separated and quantified: oligomers, oxidized triacylglycerols (OTG), diacylglycerols (DAG), monoacylglycerols (MAG) and free fatty acids (FFA). Diolein, monolein, and oleic acids were used as standards for DAG, MAG, and FFA, respectively. Since the non-oxidized triacylglycerols had been removed from the polar fractions following the AOAC Method 982.27 [14], the peak with retention time similar to triolein was taken as oxidized triacyglycerols. Components eluting before the OTG were quantified as oligomers. The contribution of specific compounds is presented as relative percentage based on the peak area.

### Free Fatty Acids (FFA)

FFA content expressed as a percentage of oleic acid was determined by AOCS Official Method Ca 5a-40 [13].

### Tocopherols

Tocopherols were analyzed following AOCS Official Method Ce 8–89 [13] using a Finnigan Surveyor HPLC (Thermo Electron Corporation, Waltham, MA) with a Finnigan Surveyor Autosampler Plus and Finnigan Surveyor FL Plus fluorescence detector, set for excitation at 292 nm and emission at 394 nm. The column was a normal-phase Microsorb 100 silica (250 × 4.60 mm; 3 μm; Varian, CA). Of each sample, 10 μL was injected and separated by mobile phase consisted of 7 % methyl-*tert*-butyl-ether in hexane with a flow rate of 0.6 mL/min. The tocopherols were quantified using external calibration for each isomer separately.

### Anisidine Value

The anisidine value (AV), a measure of secondary oxidation products, was determined according to ISO Method 6885:2004 [16].

### Color Analysis

The spectrophotometric color test of the frying oils was performed following the AOCS Official Method Cc 13c-50 [13] at 490 nm using a DU-65 spectrophotometer (Beckman, Fullerton, CA).

### Sensory Assessment of Food Fried

A 37-member consumer panel rated food acceptance on a 10 point scale where a score of 1 was assigned as bad and 10 for excellent quality [17]. Panelists were selected to represent average consumers, and the group was instructed on how to evaluate fried products. To make results more reliable panelists at every session had access to good quality fried food prepared by frying the specific product in fresh oil, used as a reference. Sensory evaluation was run daily using products from the middle of the frying cycle. Sensory evaluation was performed in a room with excellent ventilation where panelists were separated by space preventing communication. Results are presented as the combined averages, calculated for the 7 days of frying period. Calculated standard deviation illustrates the distribution of the panelists’ sensory scores.

### Statistical Analysis

Data are presented as means ± standard deviations (SD) from triplicate experiments, and each measurement was duplicated. Data were analyzed by single factor analyses of variance (ANOVA) using SPSS package (version 10.0). Statistically significant differences between means were determined by Duncan’s multiple range tests for *P* < 0.05.

## Results and Discussion

### Composition of the Oils and Products

The fatty acid and tocopherol composition of the fresh oils are listed in Table 1. The composition of CAN was typical for canola oil with oleic acid dominating and 8.5 % linolenic acid, however, HOLLCAN contained a higher amount of oleic acid (71 %) and a lower contribution of linolenic acids (Table 1). The lowest amount of linolenic acid (0.2 %) was observed in partially hydrogenated canola oil. The regular soybean oil had a typical fatty acid composition with 6.7 % of linolenic acid, and linoleic acid (53 %), twice the amount found in the hydrogenated counterpart (Table 1). As expected, the modified oils were lower in unsaturated fatty acids, particularly in the contribution of linoleic and linolenic acids (Table 1). Both hydrogenated oils have higher n-6/n-3 ratio, 10.8 and 21.6, compared to regular oils, 2.3 and 8.0 for CAN and SOY, respectively. Whereas the contributions of *trans* isomers in the hydrogenated oils were 16 and 15 times higher than the respective regular oils (Table 1). The contribution of saturated fatty acids in assessed oils was similar for both canola type oils and typical for SOY and HSOY. HCAN, however, contained lower amounts of saturated fatty acids compared to both soybean oils (Table 1).

**Table 1:** Fatty acid and tocopherol composition of frying oils (% w/w)

Fatty acids	HOLLCAN	CAN	HCAN	SOY	HSOY
C16:0	3.8 ± 0.3	4.2 ± 0.3	4.7 ± 0.4	10.2 ± 0.2	10.8 ± 0.3
C18:0	1.5 ± 0.3	1.9 ± 0.4	4.2 ± 0.3	4.4 ± 0.2	6.1 ± 0.2
C18:1	71.4 ± 0.8	60.6 ± 0.9	51.9 ± 0.7	22.6 ± 0.5	33.4 ± 0.8
C18:2	18.2 ± 0.7	19.6 ± 0.3	2.6 ± 0.2	53.6 ± 0.6	25.1 ± 0.4
C18:3	1.7 ± 0.2	8.5 ± 0.2	0.2 ± 0.1	6.7 ± 0.4	1.2 ± 0.1
C18:1*t*	0.2 ± 0.03	0.1 ± 0.02	26.7 ± 1.1	0.0	14.8 ± 1.2
C18:2*t*	0.4 ± 0.02	0.5 ± 0.02	7.9 ± 0.9	0.7 ± 0.08	6.6 ± 0.1
18:3*t*	0.4 ± 0.02	1.5 ± 0.03	0.4 ± 0.1	0.8 ± 0.06	0.9 ± 0.1
Groups and ratios of fatty acids					
Total *trans*	1.0	2.1	35.1	1.5	22.4
SAT	6.5	7.3	10.1	15.5	17.7
MUFA	73.1	62.4	53.3	22.7	33.7
PUFA	19.9	28.3	2.6	60.3	26.2
n-3	1.7	8.5	0.1	6.7	1.2
n-6	18.2	19.6	2.6	53.6	25.0
n-6/n-3	10.8	2.3	18.9	8.0	21.6
Tocopherols (μg/g)					
Alpha	270 ± 8	251 ± 5	211 ± 8	126 ± 3	79 ± 4
P8	94 ± 4	99 ± 3	84 ± 4	28 ± 2	21 ± 5
Gamma	440 ± 9	474 ± 4	367 ± 9	1428 ± 12	1,003 ± 9
Delta	9 ± 1	11 ± 1	9 ± 1	284 ± 6	383 ± 8
Total	815	837	672	1,877	1,518

*SAT* saturated fatty acids, *Oils* *HOLLCAN* High oleic low linolenic canola, *CAN* regular RBD canola, *HCAN* hydrogenated canola frying shortening, *SOY* standard RBD soybean oil, *HSOY* hydrogenated soybean frying shortening, *P8* Plastochromanol, derivative of gamma tocotrienol

*Trans* combined amount of *trans* isomers of oleic, linoleic and linolenic acids. Fatty acids with *t* represents *trans* isomers

The composition and content of tocopherols in fresh oils was typical for the specific group of oils, where soybean oils contained twice as much tocopherols as canola oils. The latter oils have twice the amount of the gamma isomer than the alpha isomer, whereas in soybean oils the gamma isomer dominated with a significant contribution of delta isomer (Table 1). Among the tested oils, HCAN contained the smallest amount of tocopherols, but with a distribution of isomers similar to typical canola oil.

Composition of lipids in fried products in Table 2 is presented. The fatty acid composition is typical for oil in which products were par-fried, French fries in HOLLCAN while fish and chicken in regular canola. Similarly to fatty acids, the composition of tocopherols also reflects oil used for par-frying (Table 2).

**Table 2:** Lipids composition of par-fried foods used in frying experiments

Fatty acids	Par-fried		
	Battered		French fries
	Chicken	Fish	
Groups and ratio			
C18:1*t*	0.68	1.42	1.53
C18:2*t*	0.53	0.33	0.48
C18:3*t*	0.65	0.49	0.54
Total *trans*	1.86	2.24	2.55
SAT	9.28	8.81	8.12
MUFA	67.37	58.61	70.27
PUFA	21.49	30.35	19.06
n-3	1.82	9.50	1.85
n-6	19.67	20.85	17.21
n-6/n-3	10.8	2.2	9.3
Tocopherols (µg/g)			
Alpha	271 ± 5	278 ± 5	321 ± 8
P8	99 ± 3	99 ± 3	94 ± 4
Gamma	494 ± 4	502 ± 4	492 ± 9
Delta	11 ± 1	11 ± 1	9 ± 1
Total	875	890	916

For abbreviations explanations see Table 1

**Fig. 1:**
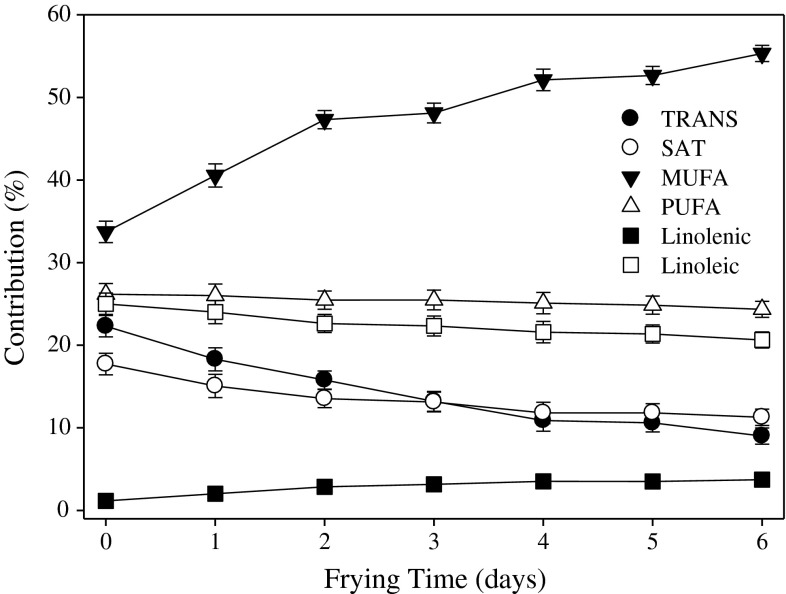
Changes of selected fatty acids in hydrogenated soybean shortening during frying products par-fried in canola oil. Results are averages from triplicate repetition of experiments where each measurement was duplicated

**Fig. 2:**
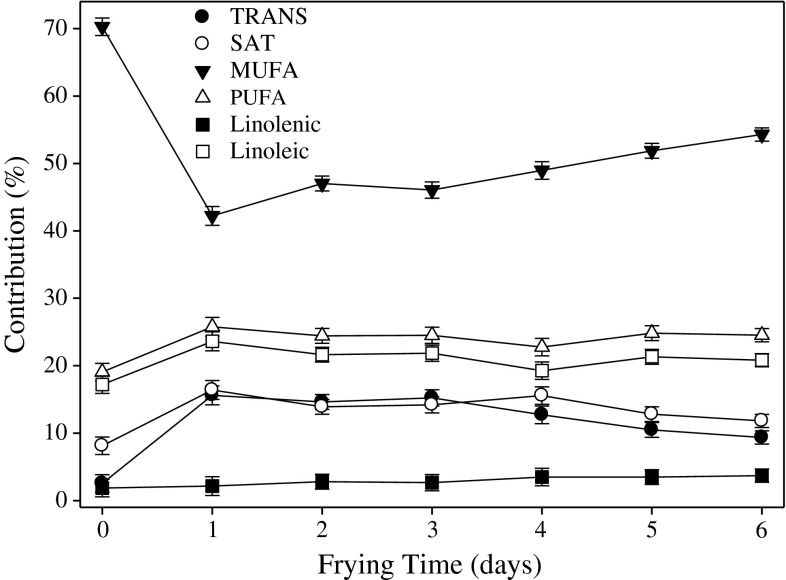
Changes of fatty acids in French fries fried in hydrogenated soybean shortening. French fries were par-fried in HOLL canola oil. For explanations see Fig. 1

Changes in the fatty acid composition of hydrogenated soybean shortening (HSOY) and in fried French fries offer a good indication of the kinetics of oil/fat exchange between frying medium and fried products (Figs. 1, 2). All the products fried in these experiments were par-fried in canola oil with the exception of French fries par-fried in HOLLCAN. Both oils had different fatty acid composition than HSOY (Table 1). This frying shortening was replaced by oil from French fries continuously during every day of frying as indicated by the changes in fatty acid composition (Fig. 1). The contribution of MUFA increased by almost 10 % after the first day of frying and continuously increased everyday up to the level close to canola oil at the end of frying. At the same time, the amount of *trans* and saturated fatty acids decreased by almost 50 % (Fig. 1).

More dramatic changes in fatty acid compositions were observed in French fries prepared in the HSOY shortening (Fig. 2). After the first day of frying the contribution of MUFA decreased from 70 to 42 % and slowly increased to 53 % over the next 5 days of frying (Fig. 2). Since soybean shortening contained high amounts of saturated and *trans* fatty acids, the amount of these groups increased significantly (*P* < 0.05) in the French fries during the 1st frying day with little or no changes thereafter (Fig. 2). Similar changes were observed for other food products assessed in this study (Data not included). These results clearly showed the dynamic of fat exchange between the frying medium and the fried product, indicating the effect of the frying oil on the nutritional quality of the prepared food.

### Polar Components (PC)

**Fig. 3:**
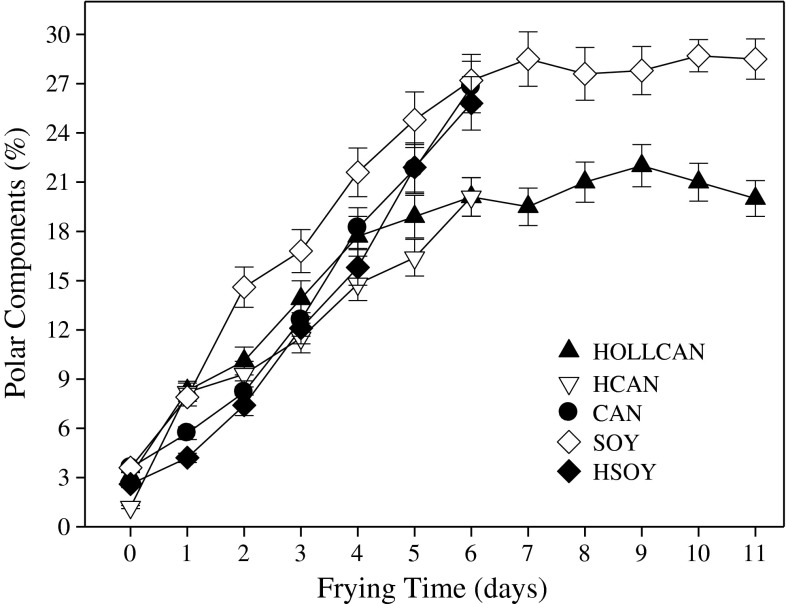
Formation of polar components during rotational frying in different oils. For abbreviations see Table 1. For Explanations see Fig. 1

Figure 3 illustrates changes in polar components during rotational frying in different oils. Measurement of polar components offers the most accurate assessment of the thermo-oxidative degradation of frying oils. Unsaturated fatty acids are among several compositional factors affecting the formation of polar compounds [7]. The rate of polar components formation was similar in all tested oils for the first 6 days of frying, subsequently the amount of polar compounds reached a plateau for SOY and HOLLCAN. This indicates that the amount of formed polar compounds was equilibrated with the degraded portion of these components, which were transformed into other compounds. Removal of polar components by fried food and replenishment with fresh oil could also contribute to the observed flattening of the PC. As can be expected, oils with elevated amounts of linolenic acid oxidized faster, producing more polar components (Fig. 3). We observed that HSOY, which had less than half of the linoleic acid compared to regular soybean oil, degraded at the same rate as the latter as measured by the formation of polar components (Fig. 3, Table 1). This indicates that the amount of this acid is still too high to improve the oil frying stability. It is also possible, however, that during processing, particularly hydrogenation, components were formed that promoted faster oxidative degradation, including residues of the catalyst. After 6 days of rotational frying, CAN, HSOY, and SOY had 27 % of polar components, which is above the discarding level set by some European countries [18]. In HOLLCAN and HCAN polar components were formed 40 % more slowly and, at the end of the 6th day of frying, the amount of these compounds was below the discarding level. At the same time, the polar compounds plateaued and stayed at the same level for the next 5 days of rotational frying in HOLLCAN (Fig. 3). Matthaus [4] found similar performances for both oils as measured by polar components formation. Warner et al. [7] stated that modified canola oils were more stable than the regular oil during frying, based on total polar component formation.

### Oligomers and Oxidized Triacylglycerols

**Fig. 4:**
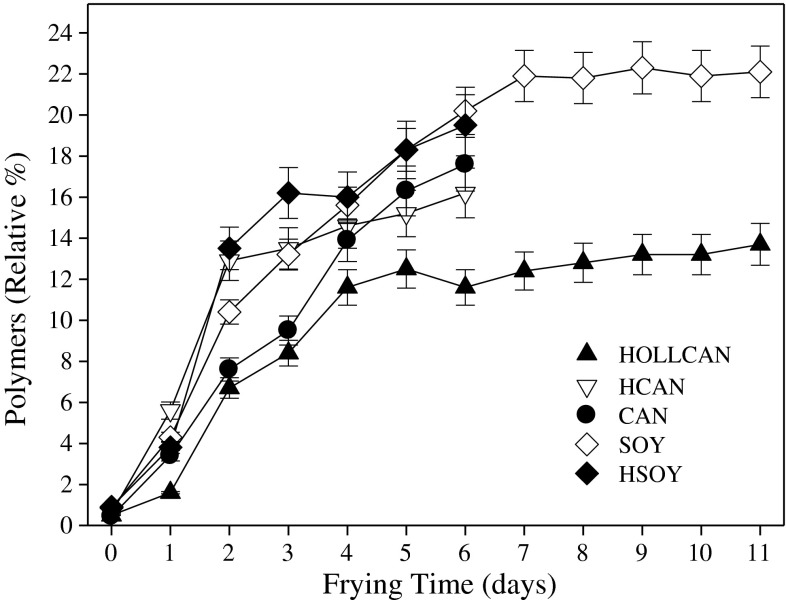
Oligomer formation during rotational frying in different oils. For abbreviations see Table 1. For explanations see Fig. 1

Oligomers are one of the products formed from oxidized triacylglycerols and are good indicators of oxidative stability of oil during frying. As a consequent of standard processing, mainly during deodorization, the amount of oligomers formed is affected by the applied temperature and time [18]. We found in freshly processed oils about 1 % of oligomers but during frying the amount of oligomers increased as the frying time expanded. Oligomers were formed faster in oils containing linoleic and linolenic acids, reaching 23 % for SOY and 14 % HOLLCAN, other oils were between those two (Fig. 4). For both soybean oils, oligomers formation was at the highest rate indicating that a certain level of linoleic acid can have a stimulating effect on thermo-oxidative degradation of oil [19]. Interestingly, canola oil with a higher amount of linolenic acid but a lower contribution of linoleic showed a significantly (*P* < 0.05) lower formation of oligomers during rotational frying than the soybean oils (Fig. 4). No significant improvement in thermo-oxidative stability as measured by oligomer formation was observed for HCAN compared to CAN despite the fact that the amount of PUFA was 11 times higher in the latter. On the other hand, HOLLCAN showed the best thermo-oxidative stability, as assessed by the lower amount of oligomers formed, irrespective of duration of frying (Fig. 4). The poorer performance of HCAN, despite the much lower amount of PUFA (2.6 %), is affected by processing, particularly hydrogenation and secondary deodorization, where some reactions leading to faster formation of oligomers are initiated. We also observed that in hydrogenated oils tocopherols degraded at a faster rate than in non-hydrogenated, causing less antioxidant available to protect oil from oxidation [6, 11].

**Fig. 5:**
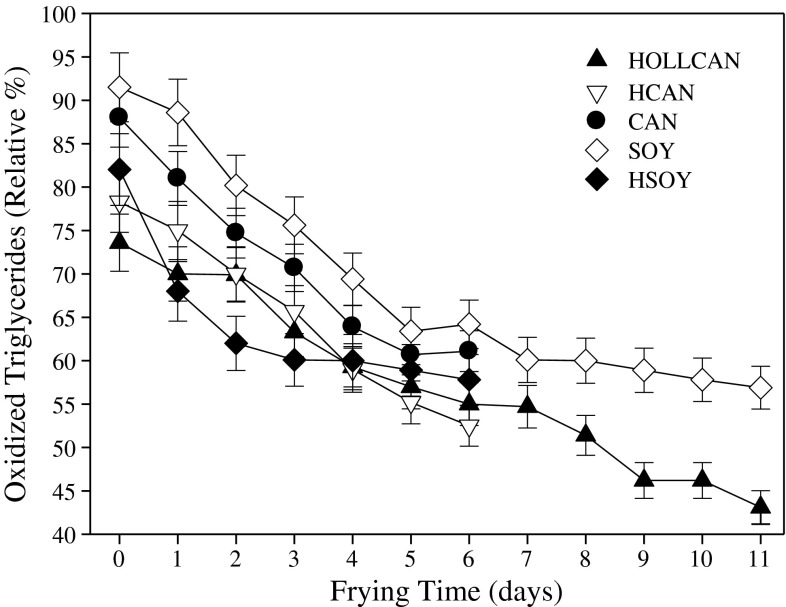
Changes in oxidized triacylglycerols during rotational frying in different oils. For abbreviations see Table 1. Measured OTG are primary oxidation products and easily decomposed into variety of secondary compounds. For explanations see Fig. 1

Oxidized triacylglycerols (OTG) are the primary products formed during PUFA oxidation and they react and are degraded further to form a host of secondary compounds, including oligomers [20]. The contribution of OTG decreased consistently over the frying period, again a faster decrease in the contribution of OTG was observed for HSOY (Fig. 5).

### Free Fatty Acids

**Fig. 6:**
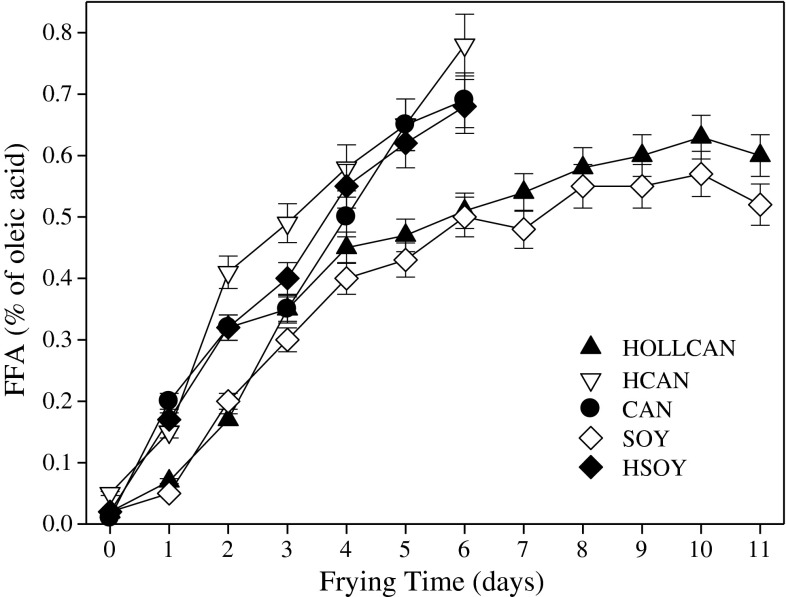
Formation of free fatty acids during rotational frying in different oils. For abbreviations see Table 1. For explanations see Fig. 1

Free fatty acids (FFA) are formed during hydrolysis of TG and as degradation products from oxidized TG (OTG). The amount of FFA increased slowly during rotational frying and did not exceed the limit of 2 % established by European regulations [22]. The content of FFA in fresh oils was lower than 0.1 %, indicating good quality processed oils. During frying in HCAN and HSOY, a faster rate of FFA formation was observed compared to the other oils (Fig. 6). Petukhov et al. [21] showed similar FFA results for HCAN where a faster rate of these components accumulation was observed than in other canola oils. Warner and Mounts [7] showed higher amounts of FFA formed during frying in SOY oil, contrary to the results from this study. Faster formation of FFA in hydrogenated oils can be stimulated by compounds formed during hydrogenation and secondary deodorization, including residue of catalyst used in the previous. Generally low amounts of FFA were formed during frying and this parameter is not suitable to be used as a quality indicator for frying oils (Fig. 6). Newly developed frying oils usually form smaller amounts of FFA than frying fats used commonly for this process [18].

### Tocopherols

**Fig. 7:**
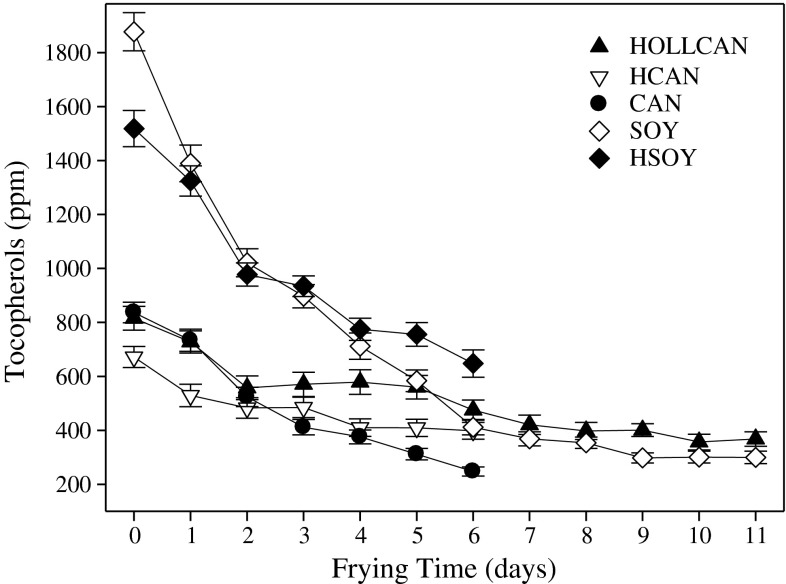
Degradation of tocopherols during rotational frying in different oils. For abbreviations see Table 1. For explanations see Fig. 1

The changes in the amount of tocopherols during frying are presented in Fig. 7. Tocopherols act as antioxidants, thus the oils in which they decay rapidly will exhibit lower oxidative stability [6]. Soybean oils showed the fastest rate of tocopherol degradation, and after 3 days of frying half of the tocopherols had disappeared, a similar half-life was observed for CAN. The slowest degradation of tocopherols was observed for HOLLCAN where, after the 6th day of frying, the amount of these components decreased by 50 %. These results are in good agreement with previous findings stating that greater oxidative stability of HOLLCAN and CAN oils appeared to be affected by slower rates of tocopherol degradation [6]. Matthaus [4] also found a slower degradation of tocopherols in HOLLCAN compared to partially hydrogenated canola oil during frying for up to 72 h. Oil with a faster degradation of tocopherols such as soybean oils resulted in a faster development of polar components during frying (Fig. 3).

Assessing relative decomposition of the individual tocopherol isomers (data not presented) we found that γ-tocopherol degraded at the fastest rate, followed by δ- tocopherol with α-tocopherol being the most stable. Gordon and Kourimska [22] showed an opposite degradation rate of tocopherols to the results of current study. However, Aggelousis and Lalas [23] and Matthaus [4] data supported the order of tocopherols decomposition established in this study.

### Anisidine Value (AV)

**Fig. 8:**
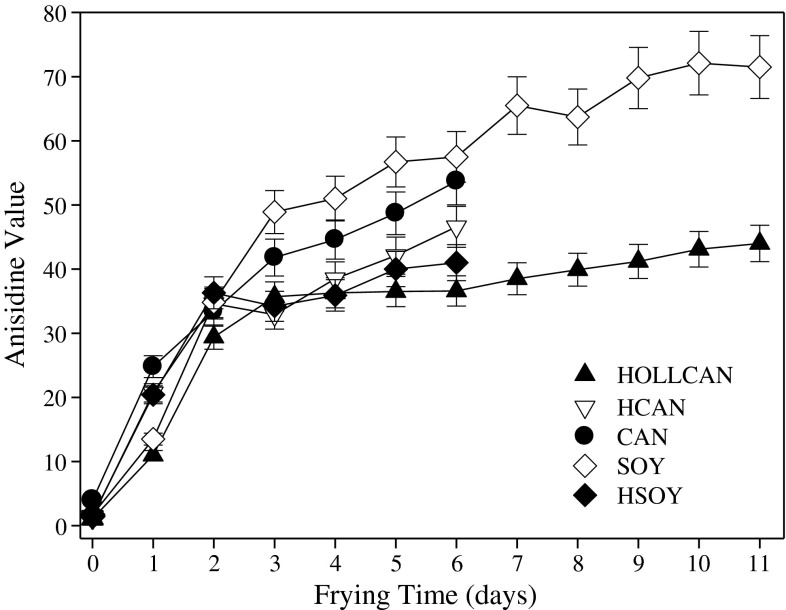
Non-volatile carbonyls formation during rotational frying in different oils. For abbreviations see Table 1. For explanations see Fig. 1

The anisidine value is used to assess relative amounts of non-volatile carbonyl compounds formed as secondary degradation products from fatty acid hydroperoxides; its changes are presented in Fig. 8. A rapid increase in AV was observed for all the oils for the first 3 days of frying, thereafter slowing down to a plateau after the 6th day of frying (Fig. 8). This leveling of the AV can be attributed to the addition of fresh oil every 2nd day of frying and transfer of these components into the fried food. HOLLCAN consistently displayed lower AV than other oils, regardless of the frying time. Conversely, the highest values were found for SOY, indicating extensive degradation of oxidized unsaturated fatty acids. The significantly higher AV for SOY compared to other oils can be attributed to its high level of PUFA, a group of fatty acids most prone to oxidative degradation. Both HCAN and HSOY tended to have lower AV than the regular ones. Those results were anticipated based on their lower amount of PUFA compared to CAN and SOY (Table 1). The data from these investigations were in good agreement with a previous study by Tompkins and Perkins [24]. On the basis of their results the hydrogenated soybean oils consistently exhibited lower AV than the non-hydrogenated one. Considering the earlier results reported by Matthaus [4], the lowest AV were found for the partially hydrogenated rapeseed oil while the high oleic rapeseed oil displayed the highest AV.

### Color Changes

**Fig. 9:**
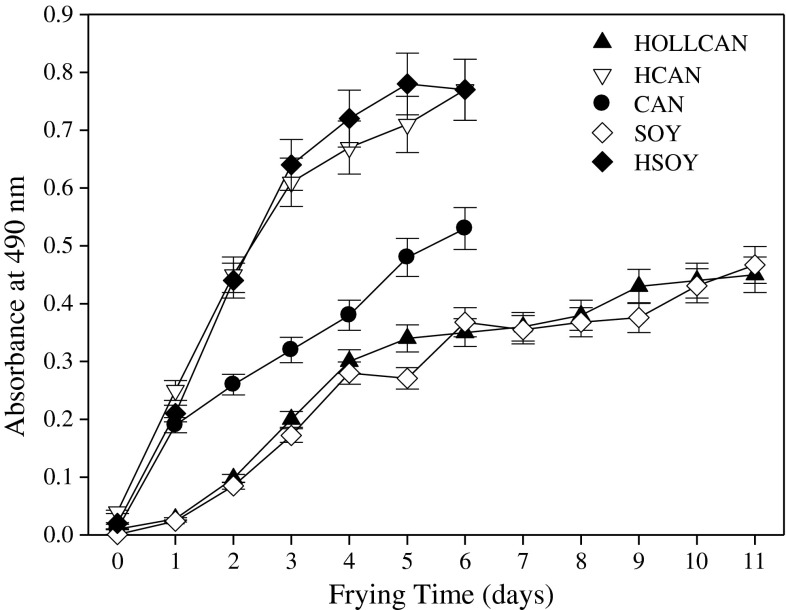
Changes in color during rotational frying in different oils. For abbreviations see Table 1

Within all the evaluated frying times, formation of the coloring components was stimulated in the oils examined (Fig. 9). HOLLCAN and SOY had significantly lower absorbance values than all of the other oils. No significant difference was observed in the rate of color component formation between HCAN and HSOY, and these two oils exhibited the highest color values at any of the measured frying times. Within the whole frying period evaluated, the average of 95 and 97 % increases in color were elicited, as measured by optical density for HCAN and HSOY, respectively. As far as CAN is concerned, the results indicated that the color at the 5th day of frying was comparable to the absorbance value at the 2 day of HCAN usage. The absorbance measured for CAN after 5 days of frying was as high as the value obtained for HOLLCAN at the 11th day of the oil usage. Darkening process of oils during frying is attributed mainly to the nonenzymatic browning compounds formation, as a result of the Maillard reaction [25]. Development of the coloring agents is used in the food industry for rapid monitoring of frying oils quality. However, it is noteworthy that the darkening of oils may also be influenced by trace pigments and tocopherols degradation in the frying oils [26].

### Sensory Assessment of Food Fried

**Fig. 10:**
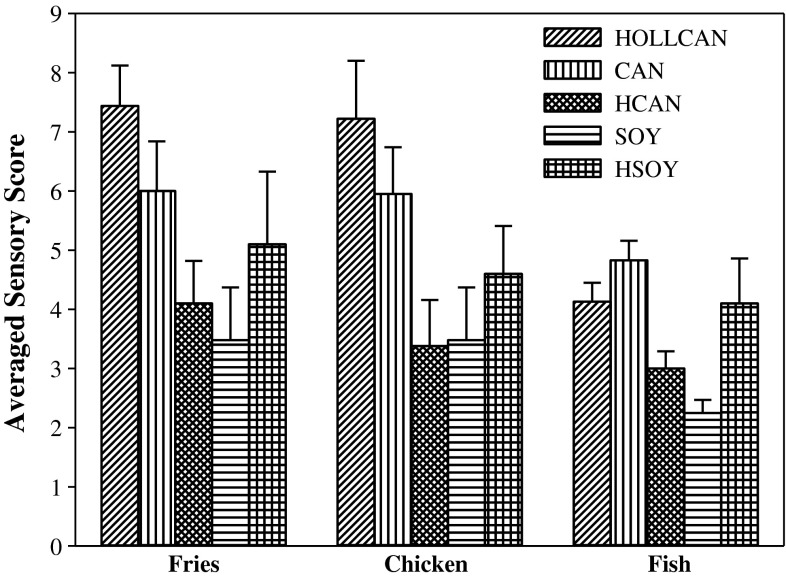
Averaged acceptance sensory scores for products fried in different oils. For abbreviations see Table 1. Scores are averages for all panelists’ scores assigned for the individual product calculated for the 7-day frying period

As far as sensory assessment is concerned, the taste, the color, the crust and the inner composition of food have profound implications. Figure 10 illustrates the results of sensory evaluation of the food products fried in selected oils. The evaluation of the French fries prepared in SOY exhibited the lowest value with the score of 3.5 compared to the other oils. Although the value obtained for HSOY (5.2) was higher than for SOY, this was not statistically significant and indicated a similar devaluation of the product. After 6 days of frying in HCAN, the French fries did appear to have a lower sensory assessment (4.2) than those fried in HOLLCAN and CAN. The products fried in both HOLLCAN and CAN with the scores of 7.5 and 6.0, respectively, were still suitable for human consumption after completion of frying. Regarding the sensory evaluation of the chicken sticks, the same trend was observed. The results indicated the apparent advantages of using HOLLCAN in the deep frying process as well as the poorest sensory assessment after frying in HCAN and SOY. The evaluation of the fish sticks exhibited lower values compared to the other products assessed with the minimum score of 2.3 reached for SOY. The scores received for HSOY and HOLLCAN were twice as high as for SOY. No significant difference was observed in the sensory scores for HOLLCAN and CAN while the product fried in HCAN exhibited a significantly lower score. After completion of frying, the fish sticks fried in CAN appeared to have the highest sensory assessment (4.8) compared to the other oils. However, all the fish sticks were found to have poor sensory acceptance, regardless of the type of oil used.

The low evaluations of the products fried in the hydrogenated oils were not surprising since it is well known that hydrogenation imparts an unpleasant characteristic flavor to food. That complex flavor, consisting of several individual odors, such as fruity, flowery or waxy, drastically decreases the quality both of the oil and the fried food [7, 26]. Contrary to results in the current study, Warner and Mounts [7] posited that the quality of the French fries prepared in hydrogenated canola oils was significantly better than those fried in a regular one. Considering the low evaluation of the products fried in SOY, the following conclusion can be drawn: undesirable soybean oil odor formed during frying (acrid, fishy, burnt and rubbery) was transferred to the flavor of food [7].

## Conclusions

High oleic low linolenic canola oil displayed the best frying life, greater than the traditionally used hydrogenated frying shortenings as indicated by the lower amounts of polar compounds, oligomers and nonvolatile carbonyl components assessed by the anisidine value. All measured factors describing frying stability of oil are direct indicators of oxidative stability and the amounts of oxidative degradation products formed. Considering the nutritional value, such as low amount of SAT and trace level of *trans* as well as high amount of oleic acid, HOLLCAN can be recommended as the currently available best solution for frying when elimination of *trans* fats and nutritional value is considered. Furthermore, HOLLCAN produced consistently fried foods with the best sensory acceptance for all frying times. Changes in fatty acid composition in French fries fried in hydrogenated soybean oil (Figs. 1, 2), provides evidence that a complete exchange of lipids between frying oil and fried product occurs. Following this evidence, the lower amounts of thermo-oxidative degradation products formed during frying in the HOLLCAN oil directly affects their amounts in fried foods.
